# Persistent Dark Cones in Oligocone Trichromacy Revealed by Multimodal Adaptive Optics Ophthalmoscopy

**DOI:** 10.3389/fnagi.2021.629214

**Published:** 2021-03-09

**Authors:** Joanne Li, Tao Liu, Oliver J. Flynn, Amy Turriff, Zhuolin Liu, Ehsan Ullah, Jianfei Liu, Alfredo Dubra, Mary A. Johnson, Brian P. Brooks, Robert B. Hufnagel, Daniel X. Hammer, Laryssa A. Huryn, Brett G. Jeffrey, Johnny Tam

**Affiliations:** ^1^National Eye Institute, National Institutes of Health, Bethesda, MD, United States; ^2^Center for Devices and Radiological Health, U.S. Food and Drug Administration, Silver Spring, MD, United States; ^3^Department of Ophthalmology, Stanford University, Palo Alto, CA, Unites States; ^4^Ophthalmology and Visual Sciences, University of Maryland School of Medicine, Baltimore, MD, United States

**Keywords:** adaptive optics, scanning laser ophthalmoscopy, optical coherence tomography, dark cones, visual function, color vision, oligocone trichromacy, pde6h

## Abstract

Dark cone photoreceptors, defined as those with diminished or absent reflectivity when observed with adaptive optics (AO) ophthalmoscopy, are increasingly reported in retinal disorders. However, their structural and functional impact remain unclear. Here, we report a 3-year longitudinal study on a patient with oligocone trichromacy (OT) who presented with persistent, widespread dark cones within and near the macula. Diminished electroretinogram (ERG) cone but normal ERG rod responses together with normal color vision confirmed the OT diagnosis. In addition, the patient had normal to near normal visual acuity and retinal sensitivity. Occasional dark gaps in the photoreceptor layer were observed on optical coherence tomography, in agreement with reflectance AO scanning light ophthalmoscopy, which revealed that over 50% of the cones in the fovea were dark, increasing to 74% at 10° eccentricity. In addition, the cone density was 78% lower than normal histologic value at the fovea, and 20–40% lower at eccentricities of 5–15°. Interestingly, color vision testing was near normal at locations where cones were predominantly dark. These findings illustrate how a retina with predominant dark cones that persist over at least 3 years can support near normal central retinal function. Furthermore, this study adds to the growing evidence that cones can continue to survive under non-ideal conditions.

## Introduction

Adaptive optics (AO) ophthalmoscopy has become increasingly used to assess cone photoreceptor structure and function in human subjects. In the normal photoreceptor mosaic, the reflectivity of individual cones is highly variable both spatially and temporally (Pallikaris et al., 2003). This reflectance signal is thought to arise from natural variations in the way light interacts with cones, including the capture, waveguiding, and backscattering of light (Roorda and Williams, 2002). However, in disease, structural and functional changes can impact the reflectivity of cones, resulting in what has been previously referred to as “dark cones.” Multimodal AO ophthalmoscopy can show cones through different contrast mechanisms simultaneously. Recently, non-confocal split detection revealed that cones with diminished or seemingly absent reflectivity in retinal disorders such as achromatopsia (Georgiou et al., 2020) can have intact inner segments even though they appear dark. However, the clinical implications and functional consequence of having dark cones remain unclear for numerous retinal diseases.

**FIGURE 1:**
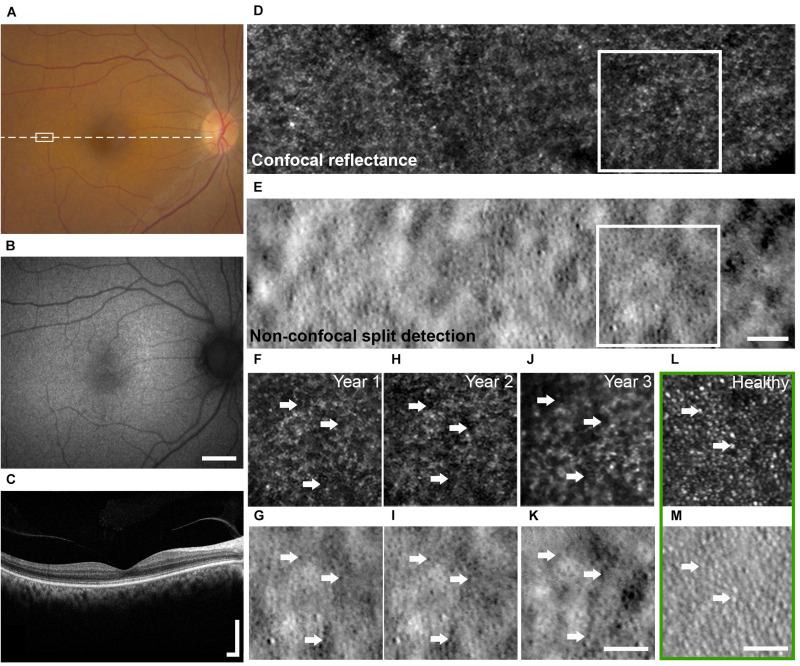
Multimodal clinical and AO imaging of a patient with OT. Color fundus photo **(A)** and fundus autofluorescence **(B)** of the right eye at baseline visit demonstrating mottled RPE with corresponding granular changes. **(C)** OCT of the macula (dashed line in **A**) showed irregularity of the IS/OS band. See Figure 5 for additional detail. Spatially and temporally co-registered multimodal AO-SLO from year 2 at white rectangle marked in **(A)** revealed the presence of dark cones with diminished cone reflectivity (**D**, confocal reflectance image) associated with intact cone photoreceptor inner segments (**E**, non-confocal split detection image). See Supplementary Figure 2 for AO images from a healthy eye for comparison. Longitudinal confocal reflectance **(F,H,J)** along with co-registered non-confocal split detection **(G,I,K)** images from the region enclosed by white boxes in **(D,E)** revealed that these dark cones persisted over a period of 3 years, and the same dark cones (e.g., arrows) seen in year 1 **(F,G)** can be tracked through year 3 **(J,K)**. AO confocal reflectance and split detection images from an age-matched healthy eye at a similar retinal location (green box) show normal-appearing cones with reflective outer segments (**L**, e.g., arrows) and their corresponding inner segments (**M**, e.g., arrows). Scale bars: **(A,B)** 2 mm, **(C)** 400 μm horizontal and vertical, **(D–M)** 50 μm.

In 1973, van Lith described a patient with a phenotype characterized by a normal fundus exam, reduced visual acuity (VA), normal or near normal color vision, normal scotopic ERG amplitudes but a much reduced photopic ERG (van Lith, 1973). He proposed the term oligocone trichromacy (OT) based on a theory of reduced cone numbers with trichromatism (van Lith, 1973), which was later confirmed in the foveal region of three OT patients using a flood illuminated AO ophthalmoscope (Michaelides et al., 2011). Here, we expand upon this earlier report by longitudinally characterizing persistent, widespread dark cones observed within and near the macula using multimodal AO scanning light ophthalmoscopy (AO-SLO) and AO optical coherence tomography (AO-OCT), alongside detailed analysis of retinal function in a patient with OT (Figure 1).

## Patients and Methods

### Study Design

The subject was a 55-year-old female born in South India to consanguineous parents, who reported a history of mildly decreased VA since childhood, minimal photophobia, and reduced contrast sensitivity. She had a history of anemia, hypertension, and chronic renal disease secondary to congenital renal hypoplasia for which she underwent successful kidney transplant. The participant provided written, informed consent. Although not a clinical trial, this research is registered on clinicaltrials.gov (Identifiers: NCT02617966, NCT01878032, and NCT02317328). National Institutes of Health Institutional Review Board (IRB)/Ethics Committee approval was obtained, and this study adhered to the tenets of the Declaration of Helsinki.

A 3-year longitudinal study with comprehensive ophthalmic examination was performed, including VA assessment, anterior segment, and dilated fundus examinations, along with AO imaging.

### Genetic Testing

DNA was extracted from whole blood of the proband for genetic testing. Initial quality control and quantification of DNA was done at the Ophthalmic Genomics Laboratory, NEI, whereas for whole exome sequencing, DNA was processed at the NIH Intramural Sequencing Center (NISC). Library preparation was done by using Roche’s NimbleGen SeqCap EZ Version 3.0 + UTR kit. Sequencing was carried out on Illumina HiSeq platform to generate 100 bp paired-end reads. Raw data were processed by using a standard in-house bioinformatics pipeline. Briefly, reads were aligned to the hg19 reference genome by using the burrows wheeler aligner and duplicates were marked with the Picard tool. Small InDels and single nucleotide variants (SNVs) were called using GATK and annotated by Variant Effect Predictor (VEP 92). By using maximum allele frequency 0.01, coding and splice variants were prioritized and then further analyzed for pathogenicity and correlation with the patient’s phenotype.

### Measurements of Retinal Function

Mesopic retinal sensitivity was measured following pupil dilation using a fundus guided perimeter (MP1, Navis Software version 1.7.6, Nidek Technologies, Padua, Italy) as previously described (Cukras et al., 2018). Briefly, retinal sensitivity was measured across 68 foci covering a 20° field centered on the fovea (Humphrey 10-2 pattern). At each locus, retinal sensitivity was measured for a 0.43° white stimulus (Goldmann size III) presented for 200 ms against a mesopic background (1.27 cd/m^2^).

Monocular color discrimination thresholds were measured along 8 axes spaced 45° apart in CIE 1976 L^∗^u^∗^v^∗^ space measured using a low vision version of the Cambridge Color Test (LvCCT) (Simunovic et al., 1998) implemented on a ViSaGe System (Cambridge Research Systems Ltd., Rochester, United Kingdom) (Zein et al., 2014). Mean color discrimination thresholds were calculated from five separate measurements along each axis at each eccentricity. The range for normal color discrimination thresholds were determined from 22 healthy volunteers. Achromatic area (AA: units = 10^6^ u^∗^v^*2^), defined by the area inside the polygon formed by the 8 chromatic discrimination thresholds was measured for foveal fixation and for two retinal eccentricities (5° and 10° along the vertical meridian from the superior retina). Color vision was also assessed with the updated Hardy-Rand-Rittler color plates (Bailey et al., 2004) and Nagel anomaloscope.

Full field ERGs (ffERGs) were recorded in accordance with the 2008 version of the International Society for Clinical Electrophysiology of Vision (ISCEV) standard (Marmor et al., 2009) as described in detail previously (Knickelbein et al., 2020). Briefly, the patient was dark-adapted for 30 min prior to the start of the ERG testing. ffERGs were then recorded from bipolar Burian-Allen contact lens electrodes (Hansen Ophthalmic Laboratories, Iowa City, IA) using a commercial electrophysiology system (LKC, Gaithersburg, MD). An Ag/AgCl electrode placed on the forehead served as ground.

### Retinal Imaging

Eyes were dilated with 2.5% phenylephrine hydrochloride and 1% tropicamide prior to retinal imaging, which included color fundus photography (Topcon, Tokyo, Japan), fundus autofluorescence (Topcon, Tokyo, Japan), optical coherence tomography (OCT, Spectralis HRA + OCT; Heidelberg Engineering, Heidelberg, Germany), and multimodal AO ophthalmoscopy.

AO retinal imaging was performed using a previously described custom instrument (Dubra and Sulai, 2011; Scoles et al., 2014) that incorporated AO-SLO [confocal reflectance (Dubra and Sulai, 2011) and non-confocal split detection (Scoles et al., 2014)] and AO-OCT [based on spectral domain OCT (Liu Z. et al., 2018)], allowing the capture of co-registered images. The system used 790 nm light (Broadlighter S-790-G-I-15-M, Superlum, Ireland) for the AO-SLO and 1080 nm light (EXS210007-01, Exalos, Switzerland) for the AO-OCT. Combined, the light levels of all sources measured at the cornea were below the maximum permissible exposure limits set by the American National Standards Institute standard Z136.1-2014. During image acquisition, the subject was asked to look at a fixation target and to blink naturally. Imaging was performed at the macula and at additional locations extending to approximately 15° in the temporal direction at the initial and subsequent visits.

### Cone Photoreceptor and Outer Retinal Length Measurements

Eye motion in AO-SLO images acquired at overlapping areas was corrected (Dubra and Harvey, 2010) after acquisition using one of the simultaneously acquired channels. Motion-corrected averaged AO images were assembled into a larger montage based on retinal features in areas of overlap. Longitudinal imaging datasets were manually overlaid and registered to non-AO retinal images. Retinal locations from both eyes at the fovea and at 5°, 10°, and 15° eccentricities in the temporal direction across the 3-year follow up period were compared, and analysis of cone photoreceptors was performed at these locations unless poor AO image quality did not permit the analysis to be done. At each location, cone identification was performed on non-confocal split detection images using custom-designed software (Liu J. et al., 2017, 2018), and cone spacing was measured based on the density recovery profile method (Ratnam et al., 2013). Dark cones were manually identified based on the transfer of cone locations determined in the split detection images onto the co-registered confocal reflectance images, and any detected cones that lacked reflective cores based on the confocal reflectance images were categorized as dark cones. At each eccentricity, cone spacing measurements were compared to previously published normative data, and the percentage of dark cones relative to all cones within a region was calculated.

Outer retinal length, defined as the distance between the inner segment (IS)/outer segment (OS) and retinal pigment epithelium (RPE) bands in OCT images, was measured using both commercial OCT and the custom AO-OCT at the same retinal locations where cone measurements were acquired. Commercial OCT was acquired with the B-scan oriented in both the temporal-nasal and the superior-inferior directions across the eye, and AO-OCT in the superior-inferior direction. Measurements on both types of OCT images in the superior-inferior direction were performed on individual A-scans after smoothing with a moving average filter in the lateral direction (kernel size: 6 pixels) and a Gaussian filter in the axial direction (window size: 5 pixels). Measurements were then averaged across all available A-scans within each B-scan.

## Results

At the time of initial evaluation, the best corrected VA of the participant was 20/32 in each eye with myopic correction. The participant had no nystagmus and her anterior segment examination was only positive for age-appropriate nuclear sclerotic and cortical cataracts. On dilated fundoscopy, she was noted to have mottled appearing RPE (Figure 1A), a few small drusenoid deposits, a healthy optic nerve, and a normal periphery. Fundus autofluorescence revealed granular hypo-autofluorescence in the posterior pole and OCT of the macula showed mild irregularity of the IS/OS junction (Figures 1B,C). Close examination of the IS/OS band in OCT suggested the presence of occasional dark gaps, which could not be explained by directionality changes due to focal elevations of the photoreceptor layer (Roorda and Williams, 2002). Over the 3-year longitudinal follow up, there were no significant clinical changes, although her cataract did progress without visual significance.

**FIGURE 2:**
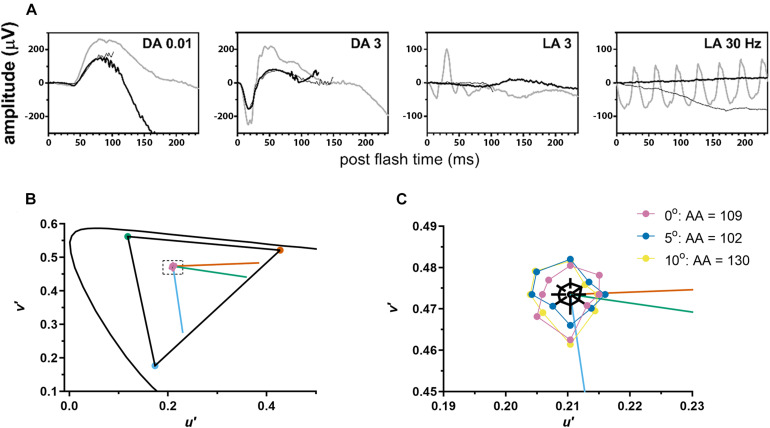
The patient demonstrated normal to near normal retinal function. **(A)** ffERG testing highlighted unrecordable photopic responses (LA, light adapted) with normal scotopic rod-mediated responses to the dim (DA0.01) flash (DA, dark adapted). Gray waveforms: age-matched healthy control; black waveforms: each eye of the patient. **(B)** Color discrimination thresholds (purple circles) were close to the white point (center of dashed rectangle) and indistinguishable from healthy volunteers on this graph. Curved line shows CIE 1976 L*u*v* space. Triangle shows the maximum gamut that could be produced by the monitor. Vermillion, green, and blue lines indicate protan, deutan, and tritan confusion lines, respectively. **(C)** A magnified view of the area inside the dashed rectangle from **(B)**. Mean color discrimination thresholds are plotted for foveal fixation (purple) and for two retinal eccentricities [5° (blue) and 10° (yellow) along the vertical meridian from the superior retina]. For each eccentricity, achromatic area (AA) for the subject with OT was slightly above the upper limit of normal. Black polygon shows mean achromatic area for healthy volunteers and black lines indicate 95% confidence intervals at each of the 8 axes.

Cone-mediated photopic ERGs were unrecordable, while rod mediated scotopic dim flash (DA0.01) responses were within the normal range (RE = 175 μV; LE = 170 μV; lower limit of normal = 167 μV) (Figure 2A). The scotopic bright flash (DA3) ERG responses which are mediated by both rods and cones were reduced 20% below the lower limit of normal. DA3 ERGs recorded to paired flashes with reduced interstimulus intervals (<20 s) were not markedly reduced, ruling out bradyopsia. Mesopic retinal sensitivity was within the normal range (≥16 dB) for 61 of the 68 loci. The remaining loci were near normal (14 dB, *N* = 6; 12 dB, *N* = 1) (Supplementary Figure 1). The participant had good color vision, making no errors on the Hardy-Rand-Rittler color plates and matching over a narrow range (32–38) on the Nagel anomaloscope. Achromatic area measured with LvCCT was slightly above the upper limit of normal (70). Additionally, achromatic area varied little with eccentricity, ranging from 109 for foveal fixation to 102 at 5° eccentricity and increasing slightly to 130 at 10° eccentricity (Figures 2B,C). Together, the fundus appearance and the functional measurements confirmed the OT phenotype.

Whole exome sequence analysis prioritized 123 SNVs and small InDels comprised of rare exonic or splice site variants. One heterozygous truncating variant NM 006205.3:c.35C > G (p.Ser12Ter) was identified in *PDE6H*, previously reported as disease-associated in homozygosity (Kohl et al., 2012; Pedurupillay et al., 2016). However, a second deleterious allele was not found. Visualization of *PDE6H* sequence reads on Integrative Genomics Viewer (IGV) did not indicate copy number of structural variations. No other deleterious variants were detected in genes associated with cone dystrophy.

**FIGURE 3:**
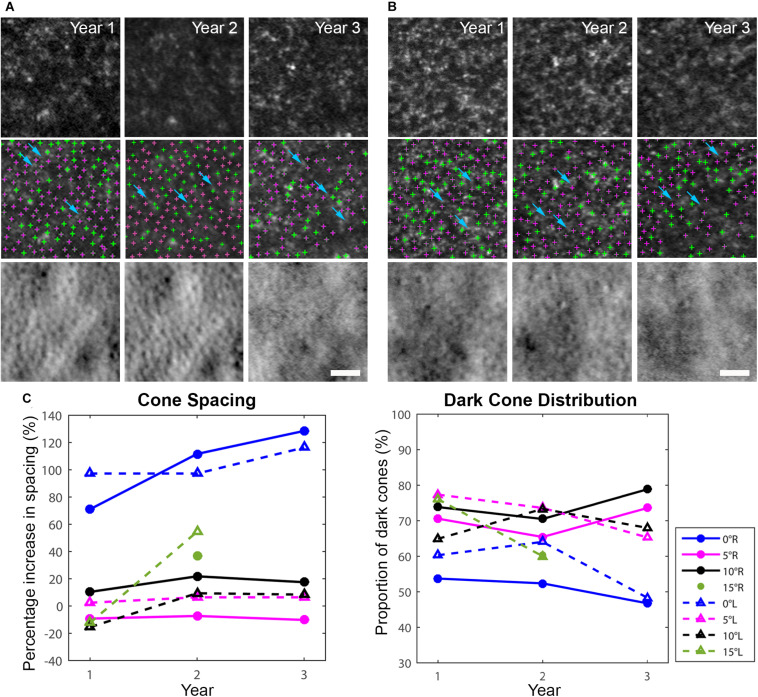
Longitudinal AO imaging of both eyes in eccentric locations (**A:** right eye, **B:** left eye) with measurements of cone spacing and dark cone distribution. Cone spacing was measured based on the density recovery profile method. **(A,B)** Dark cones in both eyes with diminished reflectivity at 5^*o*^ eccentricity across three years (top row: confocal reflectance images, middle row: confocal reflectance images with identified cones overlaid: green: normally reflective cones, magenta: dark cones), bottom row: non-confocal split detection images). A decrease in image quality was observed in year 3 mostly due to cataract progression. Examples of rods surrounding the dark cones are identified by blue arrows in **(A,B)**. Scale bars: **(A,B)** 25 μm. **(C)** Increased cone spacing was observed when compared to normative data, with the largest increase observed at the fovea (blue lines), and a large population of dark cones was found at all eccentricities. R denotes right eye, L denotes left eye.

**FIGURE 4:**
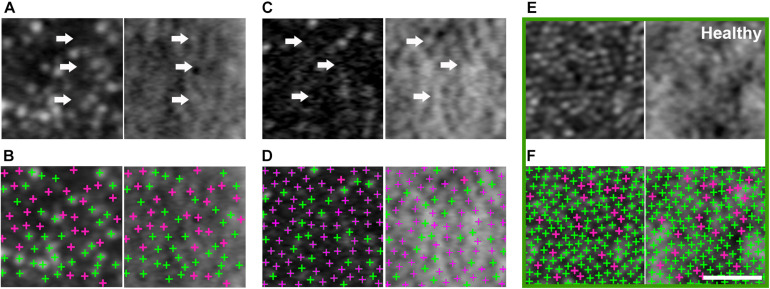
Large population of foveal dark cones observed in both eyes (**A,B**: right eye, **C,D**: left eye). Co-registered AO confocal reflectance (**A–D**: left) and non-confocal split detection (**A–D**: right) images illustrating the presence of dark cones at the fovea, which possess intact inner segments (**A,C** right: arrows) but with diminished reflectivity (**A,C** left: arrows). Multimodal AO imaging confirms that the dark areas seen in confocal reflectance (**A,C** left: arrows) are mostly due to the presence of dark cones, which are more difficult to discern in the fovea using only a single modality (i.e., **A,C** left only) due to the lack of surrounding rods (i.e., the rods in Figure 3A,B make it easier to recognize dark cones). Detected cones are identified in both confocal reflectance (**B,D**: left) and non-confocal split detection (**B,D**: right) to show the differences between normally reflective cones (green crosses) and dark cones (magenta crosses). Example foveal images from an age-matched healthy eye are shown in green box, in which normally reflective cones and higher cone density can be observed **(E,F)**. Scale bar: 20 μm.

AO-SLO imaging revealed widespread dark cones intermixed with normally reflective cones in all areas imaged during the entire longitudinal study, and the high-resolution images provided by AO-SLO allowed the same dark cones to be tracked longitudinally (Figures 1D–K, 3A,B). These dark cones can be readily identified by examining the co-registered confocal reflectance and non-confocal split detection AO-SLO images, which reveal the cone outer segment reflectivity and cone inner segment structure, respectively (Scoles et al., 2014). In a healthy eye, most of the cones are reflective (Figures 1L,M and Supplementary Figure 2). In contrast, in the patient with OT, there are numerous cones with diminished reflectivity (Figures 1F,H,J) that still retain their inner segment structure (Figures 1G,I,K), giving rise to the appearance of dark cones. Dark cones were most prevalent at eccentric locations, ranging from 51% at the fovea to 74% at an eccentricity of 10^*o*^ (Figure 3). Cone spacing measurement was performed based on identifiable cones in non-confocal split detection images (Figures 3A,B, third row; Ratnam et al., 2013). When compared to normative histological data (Curcio et al., 1990), the greatest increases in cone spacing were observed at the fovea, with either slight or no progression (i.e., increase in cone spacing) observed at eccentric locations over 3 years (Figure 3C). The large population of foveal dark cones were difficult to appreciate solely based on confocal reflectance images due to the lack of surrounding rods (Figure 4A left, Figure 4C left), but were readily visible in the matching non-confocal split detection images (Figure 4A right, Figure 4C right). In comparison, a much larger number of reflective foveal cones can be observed in a healthy eye (Figures 4E,F). Besides the presence of dark cones, lower than normal cone density was also observed across the retina. The highest cone density was at the fovea: 43,359 cells/mm^2^, within the range of expected for OT (Michaelides et al., 2011) and noticeably lower than previously reported normal value [normal histologic value (Curcio et al., 1990): 199,200 cells/mm^2^, range: 98,200–324,100 cells/mm^2^]. Cone densities at 5–15° eccentricities were 20–40% lower than the normative values and decreased slightly over time. At the final AO imaging visit, the cone density was measured to be around 12,498 cells/mm^2^ at 5° eccentricity [normal value (mean ± SD): 14,900 ± 800 cells/mm^2^ (Song et al., 2011)], 5,802 cells/mm^2^ at 10° eccentricity [normal value: 9,000 ± 900 cells/mm^2^ (Song et al., 2011)], and 3,661 cells/mm^2^ at 15° eccentricity [normal value: 8,290 ± 2,600 cells/mm^2^ (Curcio et al., 1990; Song et al., 2011)].

**FIGURE 5:**
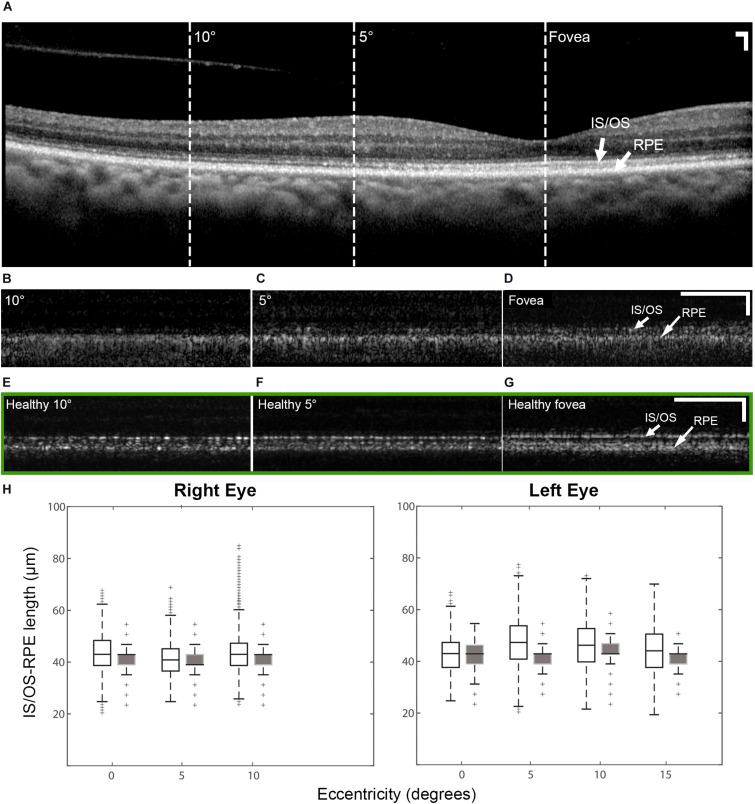
OCT of the patient right eye with outer retinal length (distance between IS/OS band and RPE) measurements and AO-OCT of a healthy right eye. **(A)** Commercial OCT of the patient acquired with the B-scan oriented in the temporal-nasal direction across the eye. Dashed lines indicate areas at which AO-OCT scans were acquired, with the B-scans oriented in the superior-inferior direction across the eye and co-registered using simultaneously acquired multimodal AO-SLO data. AO-OCT of the patient **(B–D)** revealed dark gaps in the IS/OS band, which were more pronounced at eccentric locations **(B,C)**. These dark gaps were much less apparent in an age-matched healthy eye at similar retinal locations (green box **E–G**) Scale bars: **(A–G)** 100 μm horizontal and vertical. **(H)** Box plots showing the lengths measured on AO-OCT (unfilled boxes) and commercial OCT (filled boxes) of the patient with OT. The measurements from AO-OCT were in general agreement with those from commercial OCT.

There were intermittent gaps in the IS/OS band that were detectable using commercial OCT in the patient with OT (Supplementary Figure 3). These gaps were distributed throughout the macula and were more pronounced at eccentric locations, regardless of the meridian. There were very few gaps present in the healthy eye (Supplementary Figure 3C) with normally reflective cones (Supplementary Figure 2). Although the lack of sampling and optical resolution prevented the visualization of individual cone photoreceptors in commercial OCT images (Figure 5A), similar intermittent bands which increased with eccentricity were observed using AO-OCT (Figures 5B–D), corroborating our claim that the intermittent gaps in the IS/OS band could be associated with the presence of dark cones in the retina. These gaps are especially noticeable when compared to AO-OCT of an age-matched healthy eye at similar retinal locations (Figures 5E–G). Outer retinal length derived from AO-OCT images was found to range from 43.3 ± 7.0 μm at the fovea to 43.2 ± 6.7 μm at 10° in the right eye, and 42.9 ± 6.7 μm at the fovea to 46.2 ± 8.7 μm at 10° in the left eye. These were in agreement with co-registered commercial OCT images, which measured 40.9 ± 5.2 μm at the fovea to 41.4 ± 4.4 μm at 10° in the right eye, and 41.4 ± 5.9 μm at the fovea to 43.2 ± 4.7 μm at 10° in the left eye (Figure 5H). These values were similar to measurements from both a normal subject (Liu Z. et al., 2016) and a previously reported patient with OT (Michaelides et al., 2011).

## Discussion

In this study we have investigated the possible relationship between partial loss of cone function and partial loss of cone structure in a patient with OT. The widespread presence of dark cones in OT is reminiscent of achromatopsia (Georgiou et al., 2020), especially in non-foveal regions in which dark cones can be readily identified due to the presence of surrounding reflective rods (Figures 1D, 3). Color vision testing confirmed near-normal color vision at multiple eccentricities despite the large population of dark cones (Figure 2B). The preservation of some function in a retina with a majority of dark cones is consistent with reports of both non-reflective and dysflective cones, a special case of dark cones with normal function (Bruce et al., 2015; Tu et al., 2017).

*PDE6H* (MIM 601190) is associated with autosomal recessive congenital cone dystrophy (MIM 610024) (Kohl et al., 2012; Pedurupillay et al., 2016). While a second allele was not identified, it is conceivable that this case of OT is caused by a non-coding variant altering expression of *PDE6H*, or a variant in another congenital cone dystrophy gene acting in a digenic manner, as reported in patients with achromatopsia (Burkard et al., 2018). More recently, the advent of whole genome sequencing along with RNA-seq has been very helpful in identification of a second pathogenic allele in genes causing achromatopsia, mostly deep intronic variants causing aberrant splice events (Burkard et al., 2018; Weisschuh et al., 2020). Alternative, a previously unknown gene or transcript could harbor pathogenic variation underlying this presentation.

In both eyes, there was a decrease in foveal cone density and a 104% larger than normal cone spacing, which are consistent with a VA of 20/32 (Ratnam et al., 2013). These findings confirm an earlier report of foveal cone disruption in OT using a flood illuminated AO ophthalmoscope (Michaelides et al., 2011). With the additional information provided by multimodal AO ophthalmoscopy (Dubra and Sulai, 2011; Scoles et al., 2014; Liu Z. et al., 2018), we show that most of the observed dark areas in the foveal cone mosaic are due to foveal dark cones as indicated in Figure 4, which can be difficult to recognize solely based on AO confocal reflectance images since there are no surrounding reflective rods to provide context. In addition, we show that dark cones were also present beyond the foveal region with larger population in eccentric locations (Figure 3). Spatially and temporally co-registered AO-SLO imaging confirmed that the dark cones surrounded by reflective rods (e.g., blue arrows in Figures 3A,B) seen in confocal reflectance did indeed contain intact inner segments, with a small population of cones that remained normally reflective. Further comparison of co-registered retinal regions that had both AO images and microperimetry measurements revealed that areas with reduced retinal sensitivity also had increased cone spacing (Supplementary Figures 4B,C), which was consistent with a previous publication that showed the same inverse correlation between increased cone spacing and decreased retinal sensitivity as assessed by microperimetry (Foote et al., 2019). However, there was one location with normal retinal sensitivity (20 dB) and slightly decreased cone spacing (supernormal: 10% decrease), that also had a particularly high percentage of dark cones (82%, Supplementary Figure 4D). This example suggests that some of the dark cones may still contribute to retinal sensitivity. Taken together with the functional results, the data suggest that a retina with widespread stable dark cones may not have a sufficient number of functional cones to produce a global ERG photopic response, but can still provide normal to near normal color vision and retinal sensitivity from either the few remaining normally reflective cones (fewer than 50%, Figures 3A,B, 4), or from the population of dark cones, or both. Despite the large population of dark cones, both commercial and AO-OCT imaging show that although the IS/OS band appeared to be disrupted (especially at eccentric locations; Supplementary Figure 3), outer retinal length measurements were similar to those from a healthy subject (Figure 5; Liu Z. et al., 2016). Additional measurements including more targeted testing would be required to evaluate the functional status of individual dark cones with regard to both sensitivity and color perception and to determine if the dark cones observed in OT are different than dark or dysflective cones observed in other situations (Sincich et al., 2009; Godara et al., 2012; Bruce et al., 2015; Cooper et al., 2017; Schmidt et al., 2019).

We show that the high-resolution images from multimodal AO ophthalmoscopy can identify the presence of dark cones in different retinal locations, which alongside clinical functional measurements help to shed light onto a situation in which cones may lose some, but not all aspects of their structure and function. In addition, the findings in this study further reveal the resiliency of cones. The absent ERG photopic response from this patient suggests that there was not sufficient numbers of cGMP gated ion channels that were closed in response to a light flash. Despite this, dark cones survived and persisted over a period of years and even in the presence of a large population of dark cones, the patient demonstrated normal visual acuity including normal color vision throughout the years. Importantly, this study adds to the growing evidence that cones can survive in harsh environment even with altered structure and function due to the disease (Elsner et al., 2020). We demonstrate the importance of including dark cones in quantitative cone metrics at the fovea and elsewhere for assessing variation in cell density in diseases to avoid potentially undercounting remaining cones. The combination of approaches to characterize the structure and function of cones may be useful for evaluating dark cones in OT and other disorders in which they have been described, illustrating the possibility that retinas with a majority of dark cones can retain much of their overall function.

## Data Availability Statement

The datasets presented in this article are not readily available because: the data are not publicly available due to their containing information that could compromise the privacy of research participants. Requests to access the datasets should be directed to JLi, johnny@nih.gov.

## Ethics Statement

The studies involving human participants were reviewed and approved by the National Institutes of Health Institutional Review Board. The patients/participants provided their written informed consent to participate in this study.

## Author Contributions

JLi contributed to acquisition, analysis and interpretation of data, and drafting the manuscript. TL and EU contributed to acquisition, analysis and interpretation of data, and drafting the manuscript. JLiu contributed to interpretation of data and cone analysis software development. OF contributed to acquisition, analysis, and interpretation of data. AT contributed to acquisition and interpretation of data. ZL and BB contributed to interpretation of data. AD contributed to AO-SLO instrument and DeMotion image registration software development. MJ contributed to patient referral and interpretation of data. RH, LH, BJ, and JT contributed to concept and study design, acquisition, analysis and interpretation of data, and drafting the manuscript. ZL and DH contributed to custom AO-OCT instrument and software development. All authors participated in critical revision of the manuscript for important intellectual content.

## Conflict of Interest

ZL has a patent on adaptive optics-optical coherence tomography technology and stand to benefit financially from any commercialization of the technology. The remaining authors declare that the research was conducted in the absence of any commercial or financial relationships that could be construed as a potential conflict of interest.
