# Supplementary material for: Persistent Dark Cones in Oligocone Trichromacy Revealed by Multimodal Adaptive Optics Ophthalmoscopy
Source: Front Aging Neurosci. 2021 Mar 9;13:629214. doi: 10.3389/fnagi.2021.629214 (PMC7985087; doi:10.3389/fnagi.2021.629214)
Supplement: Supplementary file 1 [file Data_Sheet_1.docx]

Supplementary Material

Persistent dark cones in oligocone trichromacy revealed by multimodal adaptive optics ophthalmoscopy

Joanne Li^1†^, Tao Liu^1†^, Oliver J. Flynn^1^, Amy Turriff^1^, Zhuolin Liu^2^, Ehsan Ullah^1^, Jianfei Liu^1^, Alfredo Dubra^3^, Mary A. Johnson^4^, Brian P. Brooks^1^, Robert B. Hufnagel^1^, Daniel X. Hammer^2^, Laryssa A. Huryn^1^, Brett G. Jeffrey^1^, Johnny Tam^1*^

^1^National Eye Institute, National Institutes of Health, Bethesda, Maryland, U.S.A.

^2^Center for Devices and Radiological Health (CDRH), U.S. Food and Drug Administration, Silver Spring, Maryland, U.S.A.

^3^Department of Ophthalmology, Stanford University, Palo Alto, California, U.S.A.

^4^Ophthalmology and Visual Sciences, University of Maryland School of Medicine, Baltimore, Maryland, U.S.A.

*** Correspondence:**Johnny Tam, Ph.D.
[johnny@nih.gov](mailto:johnny@nih.gov)

† **J. Li and T. Liu are co-first authors**

Keywords: adaptive optics ophthalmoscopy, photoreceptors, dark cones, oligocone trichromacy, PDE6H

**Supplementary Figure 1**

**
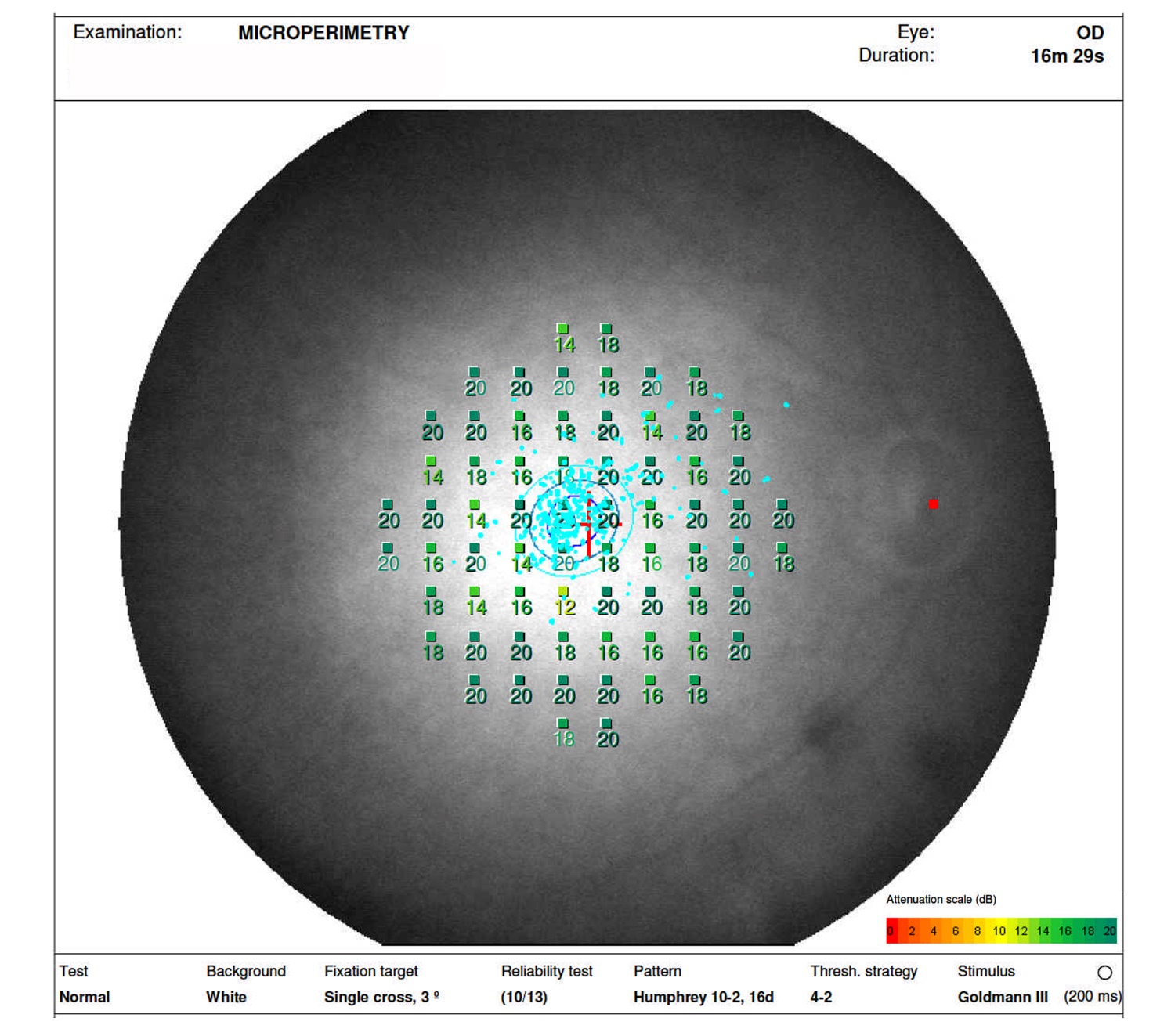
**

**Supplementary Figure 1.** Mesopic retinal sensitivity in the right eye of the patient measured following pupil dilation using a fundus guided perimeter. The measurements for the subject with OT was within the normal range (≥16 dB) for 61 of the 68 loci. The remaining loci were near normal (14 dB, N=6; 12 dB, N=1).

## Supplementary Figure 2

**
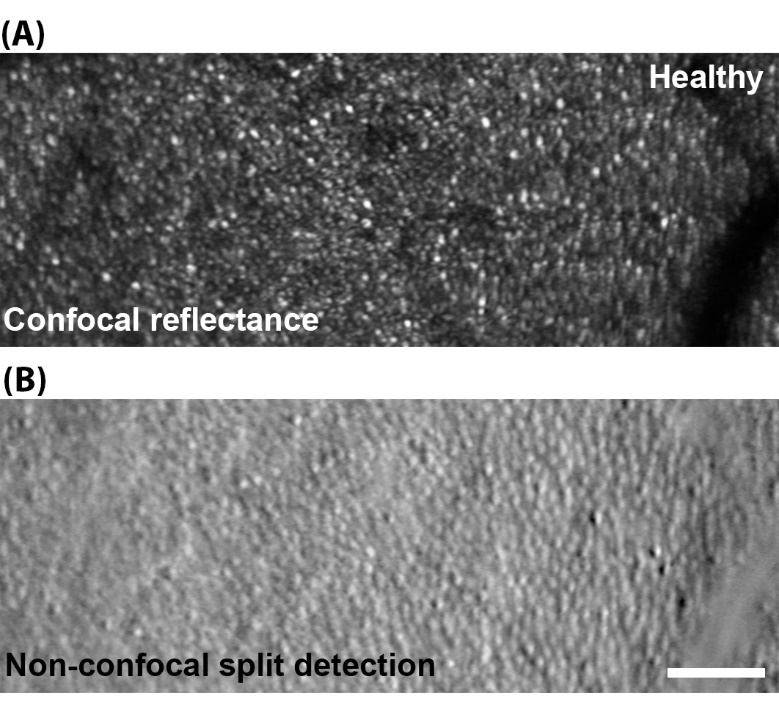
**

**Supplementary Figure 2.** Spatially co-registered AO confocal reflectance (A) and non-confocal split detection (B) images of a healthy eye for comparison with images of the patient whose retina contains large population of dark cones as shown in Figure 1D and E. Images of a healthy eye show that the majority of cone photoreceptors are reflective (i.e. there are bright dots in confocal reflectance (A) corresponding to inner segments in non-confocal split detection (B)). Scale bar: 50 μm.

**Supplementary Figure 3**


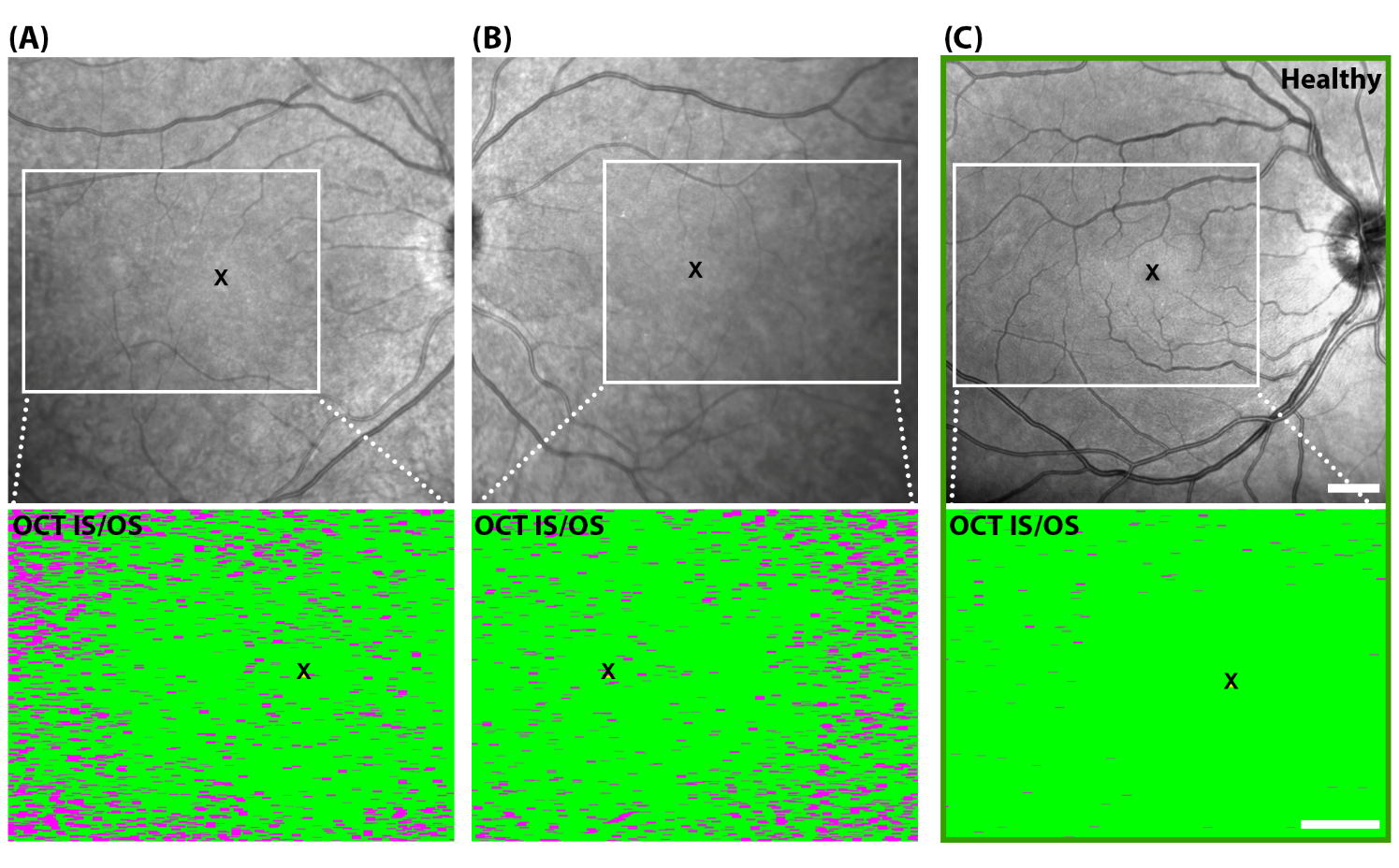


**Supplementary Figure 3.** Distribution of intermittent gaps in the IS/OS observed using commercial OCT. *En face* images showing the presence of the IS/OS band were generated by assigning a color-value to each A-scan (green: IS/OS band present; magenta: IS/OS band absent). Each pixel in the bottom row represents one A-scan (the non-square pixels are due to the A-scan interval of 5.7 µm and B-scan interval of 30 µm). There were intermittent gaps in the IS/OS present throughout the macula in the patient eyes (A: right eye, B: left eye) which were not observed in the healthy eye (C). White boxes overlaid in infrared reflectance SLO images (top row) show the regions over which the OCT volumes were analyzed, with the B-scans oriented in the superior-inferior direction across the eye. In general, gaps in the IS/OS were more noticeable at eccentric locations. The foveal locations are marked with a black X. Scale bars: 1 mm.

**Supplementary Figure 4**


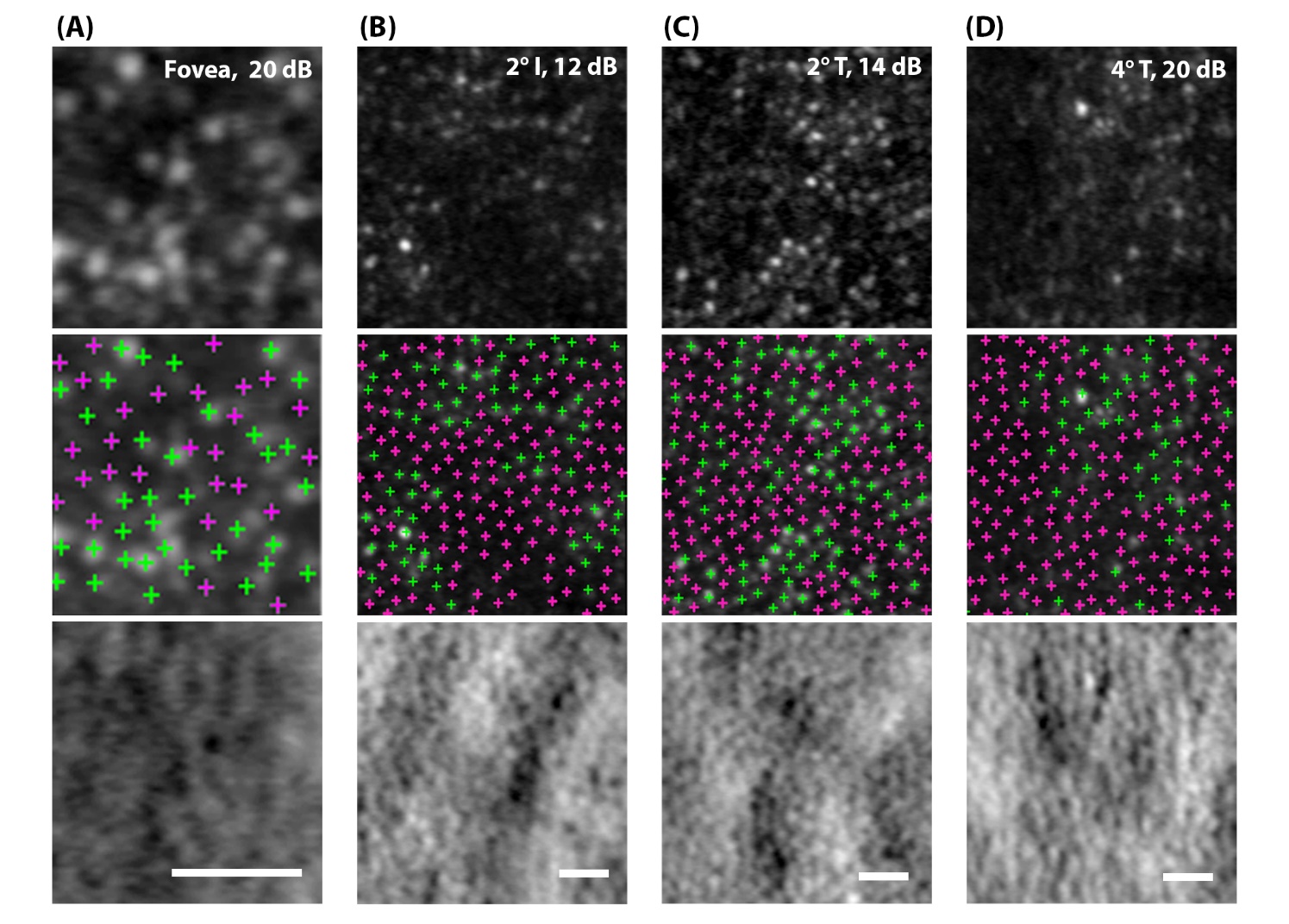


**Supplementary Figure 4.** Cone spacing and dark cone distribution measurements performed at retinal locations co-registered with microperimetry measurement locations. The two locations with lower sensitivity values (B: 2° inferior, 12 dB; C: 2° temporal, 14 dB) had a 45% and 32% increase in cone spacing, respectively, compared to the normative values. However, whereas one location with normal sensitivity had a 71% increase in cone spacing compared to the normative value (A: fovea, 20 dB; same as Fig. 4A), the other location with normal sensitivity had a 10% decrease in spacing compared to the normative value (D: 4° temporal, 20 dB). Dark cones identified using the AO images are shown in the middle row (green: normally reflective cones, magenta: dark cones). The 4° temporal location (D), which had normal retinal sensitivity (20 dB), had a dark cone density that was at least 20% higher than the other three locations (A: 54%, B: 67%, C: 64%, D: 82%), suggesting that dark cones contribute to retinal sensitivity. Scale bars: 20 μm.
